# Albumin-to-Alkaline Phosphatase Ratio is an Independent Prognostic Indicator in Combined Hepatocellular and Cholangiocarcinoma

**DOI:** 10.7150/jca.45633

**Published:** 2020-06-29

**Authors:** Feng Zhang, Shenxin Lu, Mengxin Tian, Keshu Hu, Rongxin Chen, Boheng Zhang, Zhenggang Ren, Yinghong Shi, Xin Yin

**Affiliations:** Liver Cancer Institute & Zhongshan Hospital, Fudan University, Shanghai, China; Key Laboratory of Carcinogenesis and Cancer Invasion, Ministry of Education.

**Keywords:** Albumin-to-Alkaline Phosphatase Ratio, combined hepatocellular and cholangiocarcinoma, overall survival, serum biomarker

## Abstract

**Background:** The clinical significance of Albumin-to-Alkaline Phosphatase Ratio (AAPR) has been discussed in hepatocellular carcinoma (HCC) and cholangiocarcinoma (CC). The aim of this study is to clarify the prognostic value of AAPR in patients with combined hepatocellular and cholangiocarcinoma (cHCC-CCA).

**Methods:** A total of 267 patients pathologically diagnosed as Allen type C cHCC-CCA in our institution were retrospectively enrolled and randomly divided into the training (N=187) cohort and validation (N=80) cohort. The prognostic value of AAPR was evaluated and validated. An AAPR-based nomogram was constructed and its prediction performance was assessed.

**Results:** We identified 0.43 as the optimal threshold value of AAPR by the X-tile software. In the training cohort, the median overall survival (OS) of patients with AAPR < 0.43 was significant shorter than that of those with AAPR ≥ 0.43(15.8 months vs 35 months, respectively, P < 0.001). Univariate and multivariate analyses demonstrated that AAPR was a strong indicator of OS. The concordance index (C-index), receiver operating characteristic (ROC) curves, likelihood ratio tests (LAT), Akaike information criteria (AIC) and decision curve analysis (DCA) demonstrated that AAPR outperformed the Child-Pugh (CP) grade and albumin-bilirubin (ALBI) grade in predicting OS. These findings were further verified in the validation cohort. The AAPR-based nomogram achieved C-index values of 0.76 (95%CI: 0.71-0.81) in the training cohort and 0.69 (95%CI: 0.60-0.78) in the validation cohort, which presented significant superiority to TNM stage.

**Conclusions:** Preoperative AAPR is an independent prognostic predictor in cHCC-CCA. The AAPR-based nomogram contributes to personalized prognosis prediction and clinical decision making for cHCC-CCA.

## Introduction

Combined hepatocellular-cholangiocarcinoma (cHCC-CCA) is a rare primary liver cancer showing clinical and pathological features of both hepatocellular carcinoma (HCC) and cholangiocarcinoma (CC). The incidence rate of this tumor was reported to be 0.4%-14.2% in different regions 1. Recent studies indicated that cHCC-CCA may derived from the clonal hepatic progenitor cells (HPCs) with bipotential differentiation into either hepatocytic and cholangiocytic lineages 2. In 1949, Allen and Lisa classified cHCC-CCA into three subtypes (A: double type; B: combined type; C: mixed type) 3. However, the latest 2019 WHO classification omits the subcategorization and solely defines the cHCC-CCA as the subtype with unequivocal components of both HCC and CC intimately mixed in the same mass, which was previously described as type C neoplasm by Allen and Lisa 4.

The prognosis of cHCC-CCA has been explored in previous studies. Some researchers suggested that cHCC-CCA had poorer survival outcome than both HCC and CC 5, whereas other investigations showed a better or similar prognosis of cHCC-CCA compared to CC 6, 7. Several prognostic factors for cHCC-CCA have been identified, including performance status, Child-Pugh (CP) grade, tumor size, tumor number, carcinoembryonic antigen (CEA) and carbohydrate antigen 19-9 (CA19-9) 7-10. However, these findings still remain controversial and need further validations, due to the small sample size and inconsistent pathological diagnostic criteria.

Albumin-to-Alkaline Phosphatase Ratio (AARP) is a newly developed serum biomarker-based index, which is calculated as albumin (ALB) level divided by alkaline phosphatase (AKP) level. The prognostic value of AAPR has been clarified in several malignancies including HCC 11, 12, CC 13, pancreatic ductal adenocarcinoma 14 and lung cancer 15. With regard to liver cancer, recent studies indicated that AAPR might not only reflect liver function reserve, but also associate with inflammation and cancer cell proliferation 12. However, no research has ever evaluated the prognostic value of AAPR in patients with cHCC-CCA.

Therefore, in this study, we for the first time explored the prediction performance of preoperative AAPR in patients with cHCC-CCA. Moreover, an AAPR-based nomogram was constructed to facilitate individualized prognosis prediction and clinical decision making.

## Materials and Methods

### Patients

We retrospectively screened 267 cHCC-CCA patients treated in liver cancer institute, Zhongshan Hospital between January 1993 and December 2015. The inclusion criteria were as follows: 1) pathologically confirmed as Allen type C cHCC-CCA by liver resection; 2) with CP grade A or B; 3) with detailed preoperative laboratory data and integrated follow-up data. The exclusion criteria were as follows: 1) with synchronous or previous malignancies; 2) with known renal or bone diseases. Subsequently, 267 cHCC-CCA patients were randomly divided into the training cohort (N=187) and validation cohort (N=80) in a 7:3 ratio.

The baseline and clinical characteristics of patients were collected, including age, sex, etiology, presence of underlying liver cirrhosis, laboratory results and tumor-related characteristics. The TNM stage was defined according to the eighth edition of the TNM staging system 16. The AARP was calculated by dividing the serum ALB (g/L) by serum AKP (U/L). The overall survival (OS) time was defined as the interval between the treatment date and the death date or the last follow-up date.

### Treatment and Follow-up

The surgical procedure has been described elsewhere 17. All patients were followed up every 2-3 months within the first year after hepatic resection, and thereafter, every 6 months. Physical examinations, routine blood tests, liver function tests, tumor maker tests were practiced routinely. Radiological examinations including chest radiography, computerized tomography (CT) or magnetic resonance imaging (MRI) were performed to screen tumor recurrence.

### Statistical Analysis

Continuity variables were expressed as mean with standard deviation (SD) and compared by student's t test. Categorical variables were described as number with percentages and compared by either Pearson χ^2^ analysis or Fisher's exact test.

The optimal threshold value of AAPR was determined by the X-tile statistical software (version 3.6.1, Yale University, New Haven, CT, USA) 18 regarding to OS. Patients were divided into either the low-AAPR or high-AAPR group according to the defined cut-off value. Kaplan-Meier analysis was employed to estimate the survival curves and log-rank test was used to examine the survival differences. Univariate analysis was performed to determine significant variables associated with OS. Variables with P value < 0.1 were further included in multivariate Cox proportional hazards regression model. The predictive performances of AAPR, CP grade and albumin-bilirubin (ALBI) grade were compared by using the concordance index (C-index) values, area under the curves (AUCs), likelihood ratio tests (LAT) and Akaike information criteria (AIC). In addition, decision curve analysis (DCA) was performed evaluating the net benefit of different liver function indices by using R package rmda 19. The AAPR-based nomogram for predicting 1-, 2- and 3-year OS rates was constructed based on independent prognostic factors identified by multivariate analysis. The calibration plots, C-index, AUCs, LAT and AIC were employed to assess the predictive value of the model in comparison with the 8th edition of American Joint Committee on Cancer TNM staging (AJCC TNM-8).

All statistical analyses were performed by using Stata software (version 15.1, StataCorp, College station, TX) and R software (version 3.5.1). A two-tailed P value of less than 0.05 was considered as statistically significant.

### Ethical Statement

This study was approved by Medical Ethics Committee of Zhongshan Hospital affiliated to Fudan University (No: B2019-169) and complies with the Helsinki Declaration.

## Results

### Baseline clinical characteristics

The main demographic and clinical characteristics of the training cohort and validation cohort were shown in Table 1. In the training cohort, there were 146 (78.1%) male and 41 (21.9%) female patients, with a mean age of 52.6 ± 11.7 years. 141 (75.4%) patients had a background of hepatitis B virus (HBV) infection. 131 (70.1%) patients had liver cirrhosis and 156 (83.4%) patients exhibited CP grade A. As for laboratory results, the mean level of ALB and AKP were 41.4 ± 4.3 g/L and 93.9 ± 37.8 U/L, respectively. And Alfa-Fetoprotein (AFP), CEA and CA19-9 were positive in 103 (55.1%), 29 (15.5%) and 59 (31.6%) patients, respectively. As for the tumor characteristics, there were 96 (51.3%) patients with tumor diameter larger than 5 cm and 64 (34.2%) patients with multiple tumors. Macroscopic vascular invasion (MVI) and lymph node involvement (LNI) were detected in 17 (9.1%) and 18 (9.6%) patients, respectively. The number of patients classified as AJCC-TNM Ⅰ, Ⅱ, Ⅲ and Ⅳ were 83 (44.4%), 35 (18.7%), 47 (25.1%) and 22 (11.8%), respectively.

### Clinicopathological characteristics of patients in the low-AAPR group and high-AAPR group

The optimal threshold value of AAPR was determined as 0.43 based on X-tile analyses in the training cohort (Figure S1). Subsequently, patients were dichotomized into either low-AAPR group (AAPR < 0.43, N=75) or high-AAPR group (AAPR ≥ 0.43, N=112). The correlations between AAPR and other clinicopathological features were shown in Table 2. Patients with low-AAPR levels had older age, poorer liver function (CP grade B or ALBI grade 2 & 3) and higher levels of γ-glutamyl transpeptidase (γ-GT), CEA and CA19-9 (P < 0.05). No significant associations were found between AAPR and gender, etiology, liver cirrhosis, alanine aminotransferase (ALT), total bilirubin (TB), AFP, tumor characteristics and TNM stage.

### Survival analyses

The mean follow-up duration was 25.2 (Range: 1-227) months. At the end of follow-up, 147 (55.1%) patients died and 112 (42.0%) patients showed tumor recurrence. In the training cohort, the median OS time was 29 (95%CI: 18-36) months and the 1-, 2- and 3-year OS rates were 70.8%, 52.0% and 40.1%, respectively. In the validation cohort, the median OS time was 24 (95%CI: 18-36) months and the 1-, 2- and 3-year OS rates were 79.3%, 48.1% and 36.6%, respectively. No significant survival differences were found between the training cohort and validation cohort (P = 0.972).

**Figure 1:**
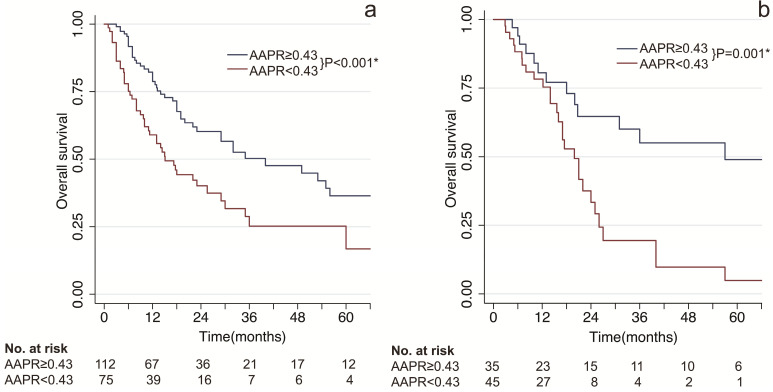
Kaplan-Meier survival estimates between subgroups by AAPR. (**a**) overall survival by AAPR in the training cohort; (**b**) overall survival by AAPR in the validation cohort. AAPR: albumin-to-alkaline phosphatase ratio. * statistically significant.

As shown in Figure 1, patients with low AAPR levels presented significant poorer survival compared to patients with high AAPR levels. In the training cohort, the median OS time of patients with low AAPR and high AAPR levels were 15.8 months vs 35 months, respectively. And the 1-, 2- and 3-year OS rates were 70.8%, 52.0%, 40.1% in the low-AAPR group, 79.3%, 48.1%, 36.6% in the high-AAPR group, respectively (Figure 1A). Furthermore, these findings were also verified in the validation cohort (Figure 1B).

### Univariate and multivariate analyses

In the training cohort, univariate analysis suggested AAPR, CP grade, γ-GT, CEA, CA19-9, tumor size, tumor number, MVI, LNI and TNM stage were significantly associated with OS. By further multivariate analysis, AAPR, γ-GT, CEA, CA19-9 and TNM stage were identified as significant independent prognostic factors. While in the validation cohort, univariate and multivariate analyses confirmed that AAPR and TNM stage were strong indicators of OS in patients with cHCC-CCA (Table 3).

### Prognostic prediction performance of AAPR in comparison with different liver function assessment methods

**Figure 2:**
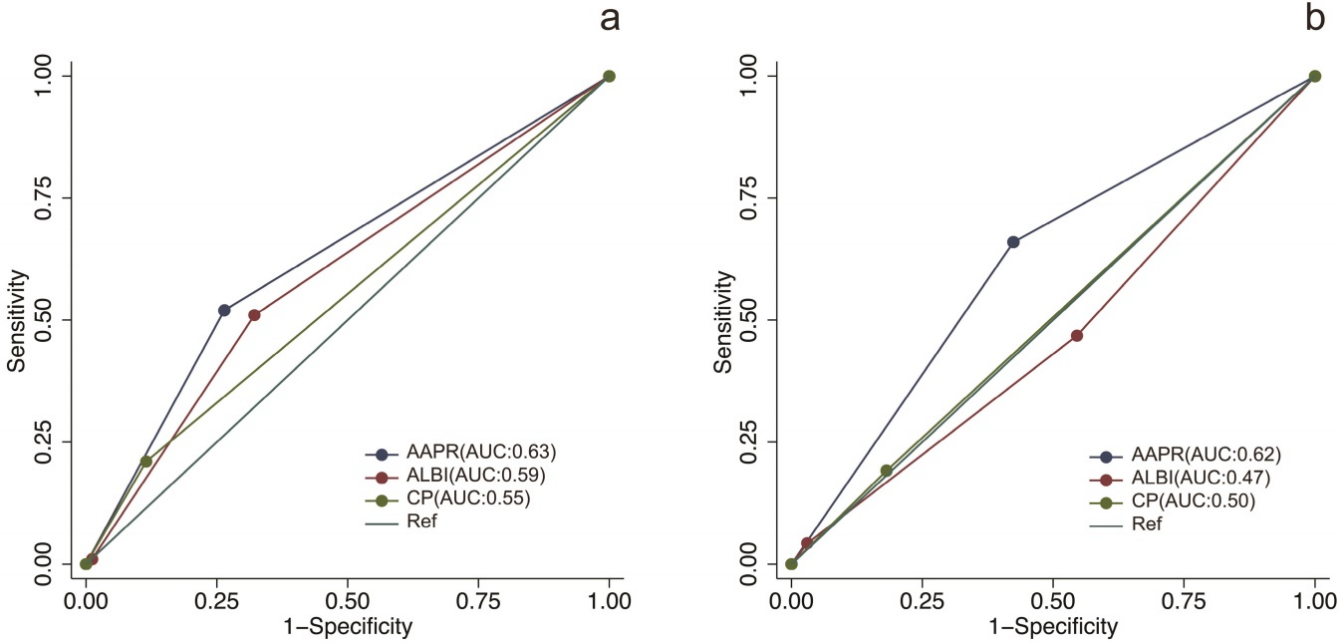
The ROC curves of AAPR, CP grade and ALBI grade for predicting overall survival in the training cohort and validation cohort. (**a**) ROC curves in the training cohort; (**b**) ROC curves in the validation cohort. ROC, receiver operating characteristic; AAPR: albumin-to-alkaline phosphatase ratio; CP grade: Child-Pugh grade; ALBI grade: albumin-bilirubin grade.

**Figure 3:**
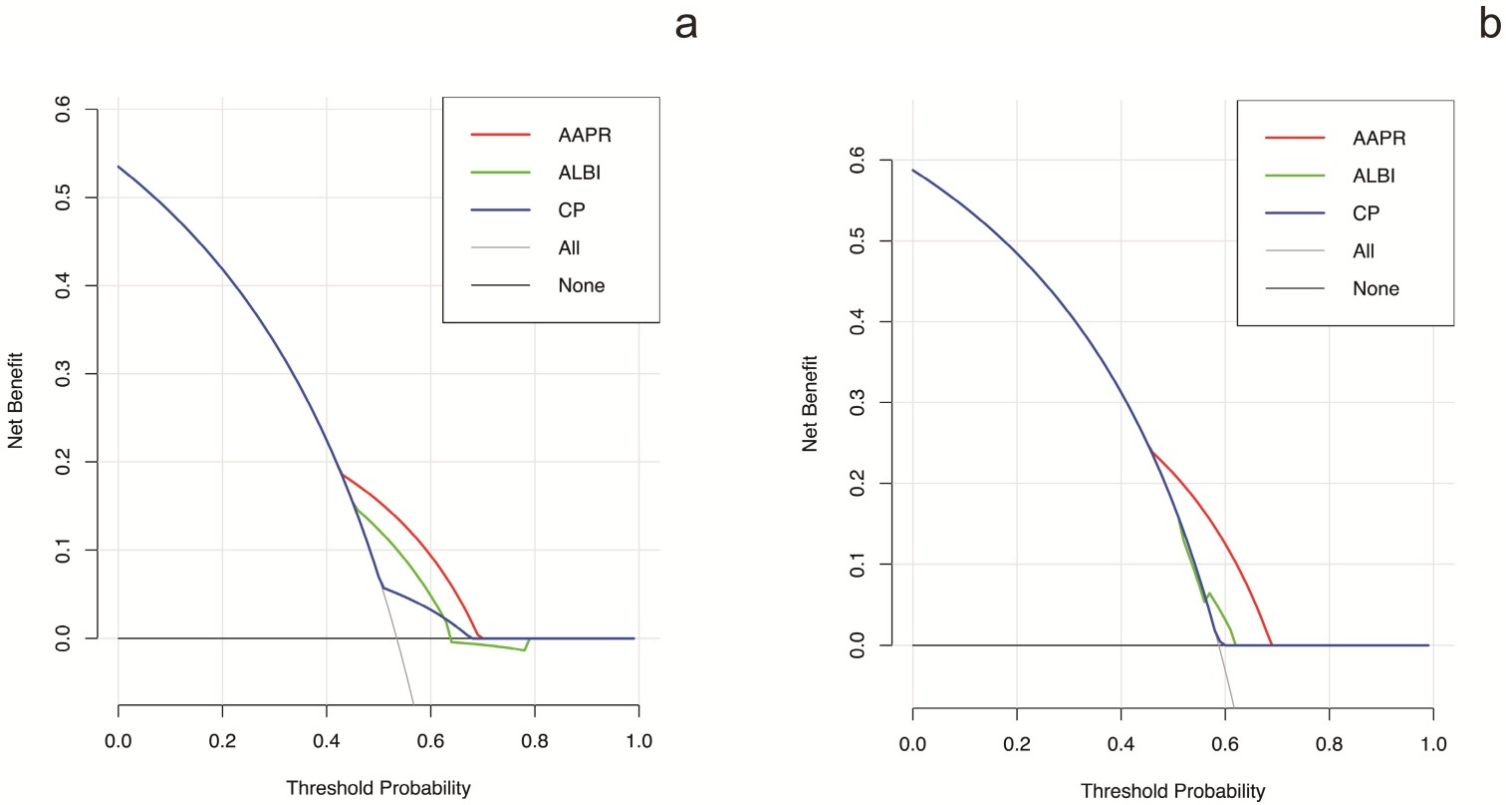
DCA for OS of different liver function assessment methods. (**a**) DCA for OS in the training cohort. (**b**) DCA for OS in the validation cohort. DCA: decision curve analysis; OS: overall survival.

The prognostic prediction performances of AAPR, CP grade and ALBI grade were evaluated by using C-index, AUCs, LRT and AIC (Table 4, Figure 2). In the training cohort, the C-index values of AAPR, CP grade and ALBI grade were 0.61 (95%CI: 0.55-0.67), 0.57 (95%CI: 0.52-0.62) and 0.56 (95%CI: 0.50-0.62), respectively. The AUCs of AAPR, CP grade and ALBI grade were 0.63 (95%CI: 0.56-0.70), 0.59 (95%CI: 0.52-0.66) and 0.55 (95%CI: 0.50-0.60), respectively. In addition, AAPR had the largest LAT χ^2^ of 11.7 and lowest AIC value of 854, which outperformed CP grade and ALBI grade. In the validation cohort, AAPR also displayed the largest C-index value, AUCs, LAT χ^2^ value and lowest AIC value. Furthermore, DCA demonstrated that AAPR had superior clinical usefulness compared to CP grade and ALBI grade (Figure 3). All these findings suggested AAPR could serve as a better prognostic predictor in comparison with CP grade and ALBI grade in cHCC-CCA.

### The AAPR-based nomogram and its predictive value

**Figure 4:**
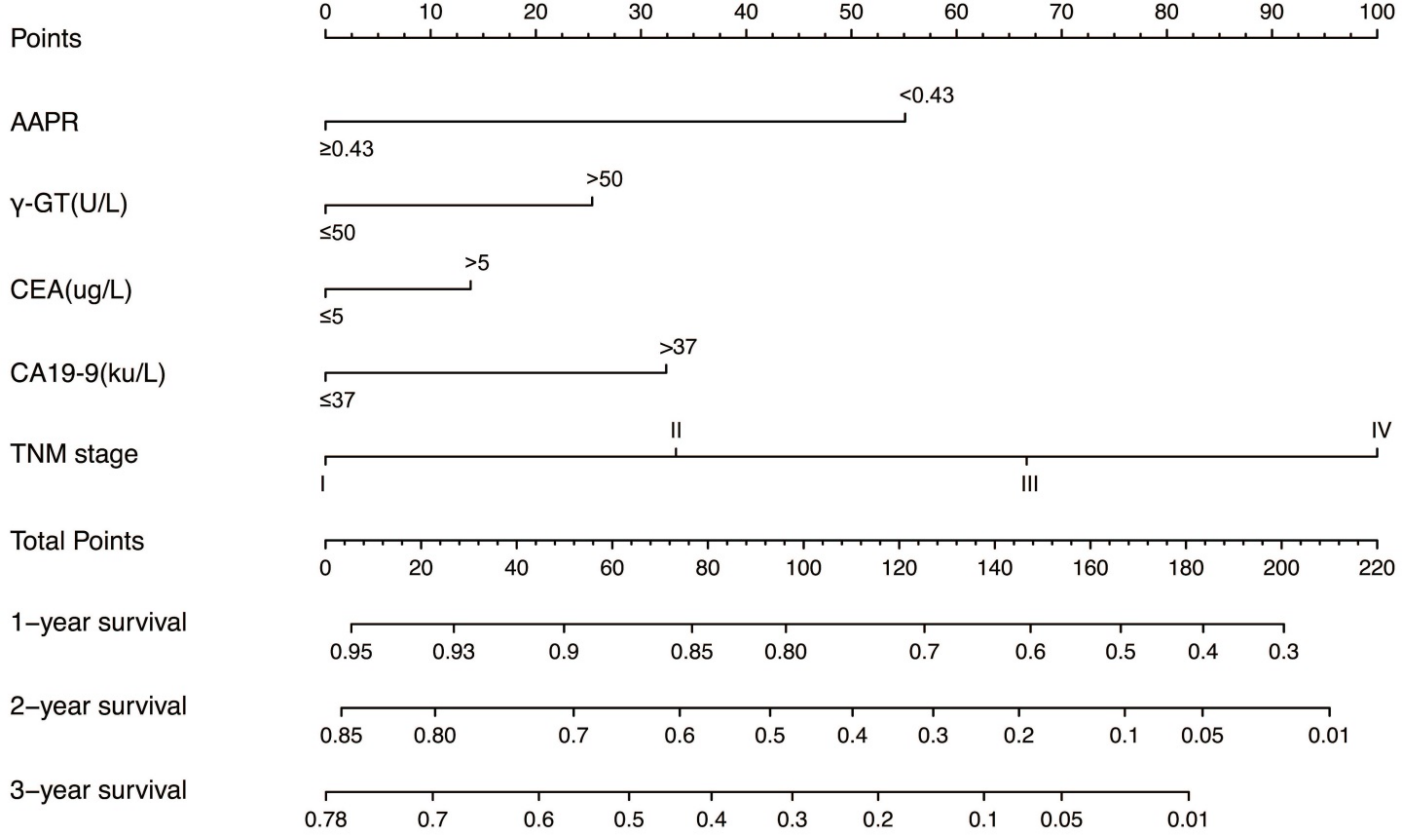
The AAPR-based prognostic nomogram for cHCC-CCA patients. AAPR: albumin-to-alkaline phosphatase ratio; γ-GT: γ-glutamyl transpeptidase; CEA: carcinoembryonic antigen; CA19-9: carbohydrate antigen 19-9.

**Figure 5:**
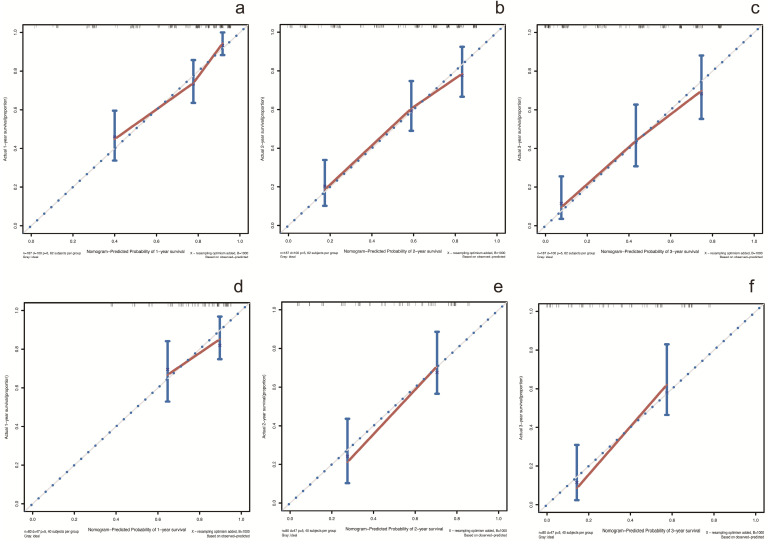
Calibration curves for predicting 1-, 2- and 3-year OS in patients with cHCC-CCA in the training cohort and validation cohort. (**a-c**) calibration curves for predicting 1-, 2- and 3-year OS in the training cohort; (**d-f**) calibration curves for predicting 1-, 2- and 3-year OS in the validation cohort. OS: overall survival.

In the training cohort, by using multivariate Cox proportional hazards model, 5 variables were identified as independent prognostic factors, including AAPR, γ-GT, CEA, CA19-9 and TNM stage. The nomogram which predicted 1-, 2- and 3-year OS was constructed based on the 5 variables and each variable was assigned a score according to their β coefficients (Figure 4). The calibration plots in both training and validation cohorts proved the excellent predictive value of the AAPR-based nomogram (Figure 5). Moreover, the AAPR-based prognostic nomogram significantly outperformed TNM stage by using C-index, AUCs, LRT and AIC in both training and validation cohorts (Table 5).

## Discussion

cHCC-CCA is a distinct form of primary liver cancer showing features of both HCC and CC 20. Due to the low incidence of cHCC-CCA, there is still a lack of investigation on prognostic prediction in cHCC-CCA. In present study, we for the first time identified the prognostic value of AAPR in patients with cHCC-CCA.

AAPR at 0.43 was determined as the optimal cut-off value in present study. cHCC-CCA patients with AAPR < 0.43 were divided into low-AAPR group, whereas patients with AAPR ≥ 0.43 were divided into high-AAPR group. Patients with low levels of AAPR had significantly unfavorable prognosis in both training and validation cohorts (P < 0.001 & P = 0.001, Figure 1). Moreover, based on univariate and multivariate analyses, AAPR was identified as an independent prognostic factor in the training cohort as well as validation cohort (P = 0.007 & P = 0.034, Table 3). These results collectively indicated that preoperative AAPR was an important prognostic predictor in cHCC-CCA.

ALB and AKP is the two basic parameter incorporated into AAPR, which are easily accessible and relatively inexpensive routine laboratory indices. AKP is a hydrolase enzyme with multiple isoforms that is mainly expressed in liver, bile duct and bone, etc. 15. It has been proposed that certain pathological conditions including biliary cirrhosis, tumorigenesis of HCC or CC and liver injury may increase AKP levels 21, 22. Although the prognostic value of AKP has been scarcely discussed in cHCC-CCA, it has been identified as a prognostic indicator for HCC, CC 23, 24 and other malignancies 25, 26. Regarding the underlying mechanisms, an in vitro study showed that high AKPase activities in the nucleolus may be related to high levels of proliferation of tumor cells 27. Some researchers also identified the association between AKP and epithelial mesenchymal transition (EMT) in HCC 28, 29. In addition, it's reported that AKP can be an indicator of oxidative stress, which plays a major role in inflammation 15. Therefore, in primary liver cancer, AKP is an index which not only associates with liver function, but also links to tumor proliferation, invasion and inflammation status. ALB is an important index in CP grade. It's reported that ALB can predict survival in several malignancies including HCC, CC, colorectal cancer and renal cell carcinoma, etc. 30-32. Moreover, serum ALB serves as a nutritional index and reflects synthetic function of liver. Low levels of ALB can result in impairment of human immunity and eventually contribute to poor prognosis 33. Investigations proposed that ALB also modulated inflammatory reaction, which plays a major role in tumorigenesis 34. In addition, a recent research showed that ALB suppressed the proliferation of HCC cells directly by increasing the G0/G1 cell population 35.

To the best of our knowledge, the CP grade and ALBI grade are the most widely used assessment tools for liver function reverse. Regarding CP grade, it has been set as an important index in several staging systems including the Barcelona Clinic Liver Cancer (BCLC) staging system 36, the Cancer of the Liver Italian Program (CLIP) score 37 and the Japan integrated staging (JIS) score 38. However, the CP grade was initially derived from patients with liver cirrhosis, and cirrhosis is not always accompanied with cHCC-CCA 20. Our previous study has identified that cHCC-CCA is a liver malignancy with lower incidence of cirrhosis in comparison with HCC 39. Moreover, CP grade incorporates two highly subjective indices of ascites and hepatic encephalopathy, which compromises its prediction performance in liver malignancy. ALBI grade, developed based on a large cohort of HCC patients, is a more objective method for liver function evaluation 40. Although ALBI grade has showed good performance in HCC patients, its discrimination efficacy in cHCC-CCA has not been confirmed. In present study, unfortunately, both CP grade and ALBI grade failed to display prognostic values in cHCC-CCA based on multivariate analysis. In contrast, AAPR was identified as an independent predictor of prognosis in the training cohort as well as the validation cohort (Table 3). Moreover, the C-index, AUCs, LAT χ^2^ and AIC demonstrated that AAPR had a better discrimination efficacy in comparison with CP grade and ALBI grade in cHCC-CCA (Table 4, Figure 2). DCA based on net benefit and threshold possibilities also demonstrated that AAPR may achieve better clinical usefulness compared with CP grade and ALBI grade (Figure 3).

To facilitate prognosis prediction in cHCC-CCA patients, an accurate prognostic model is in utmost need. To date, the most widely accepted prognostic system for cHCC-CCA is the AJCC TNM system 16. However, TNM system only takes tumor characteristics into account and lacks liver function indices, which compromised its predictive performance in liver malignancies with background hepatitis or liver cirrhosis. To address this problem, we constructed a nomogram based on 5 variables including TNM stage, AAPR, γ-GT, CEA and CA19-9 (Figure 4). The AAPR-based prognostic nomogram achieved C-index and AUC values of 0.76 (95%CI: 0.71-0.81) and 0.77 (95%CI: 0.70-0.83) in the training cohort and of 0.69 (95%CI: 0.60-0.78) and 0.74 (95%CI: 0.63-0.86) in the validation cohort, which significantly outperformed the TNM stage. The LAT and AIC values also supported this finding (Table 5).

Surgical resection is the only curative option for localized cHCC-CCA 20. Several recent studies showed postoperative TACE 41-43 and chemotherapy 7, 44, 45 may be effective adjuvant treatments for cHCC-CCA undergoing surgical treatment. Therefore, with the AAPR-based nomogram, clinicians can identify patients with poor survival outcome after liver resection. For these patients, the adjuvant treatments such as TACE and chemotherapy are recommended. Moreover, cHCC-CCA patients with poor prognosis should be followed up more intensively after surgery.

However, there are some limitations in this study. First, this is a retrospective research conducted in a single center, which may cause selection bias. Second, due to the rarity of cHCC-CCA as well as incomplete records in publicly available databases, external validations were not performed yet. Third, the threshold value of AAPR in present study was determined by the X-tile software, no consensual cut-off value has been proposed yet. Fourth, AAPR should be employed with full consideration of concurrent malignancies, bone and renal diseases.

In summary, our study identifies that the albumin-to-alkaline phosphatase ratio is an independent predictor of prognosis in cHCC-CCA. Based on this finding, we develop an AAPR-based nomogram for prognosis prediction and further decision making in cHCC-CCA. Future external validation studies and prospective researches from different cohorts are still warranted.

## Supplementary Material

Supplementary figure S1.

## Figures and Tables

**Table 1:** Characteristics of the training and validation cohorts

Characteristics	Total (N=267)	Training cohort (N=187)	Validation cohort (N=80)	P value
Age (years)	52.3±11.6	52.6±11.7	51.5±11.3	0.466
**Sex**				0.326
Male	204 (76.4)	146 (78.1)	58 (72.5)	
Female	63 (23.6)	41 (21.9)	22 (27.5)	
**Etiology**				0.515
HBV infection	196 (73.4)	141 (75.4)	55 (68.8)	
HCV infection	5 (1.9)	3 (1.6)	2 (2.5)	
Other	66 (24.7)	43 (23.0)	23 (28.8)	
**Liver cirrhosis (yes)**	186 (69.7)	131 (70.1)	55 (68.8)	0.832
**ALT (U/L)**	44.6±56.4	41.4±48.5	52.0±71.5	0.160
**ALB (g/L)**	41.4±4.5	41.4±4.3	41.5±4.9	0.881
**TB (umol/L)**	15.7±24.0	13.8±14.3	20.0±37.7	0.053
**AKP (U/L)**	101.6±89.4	93.9±37.8	119.7±151.8	0.030
**γ-GT (U/L)**				0.432
≤50	103 (38.6)	75 (40.1)	28 (35.0)	
>50	164 (61.4)	112 (59.9)	52 (65.0)	
**AARP**	0.50±0.22	0.51±0.21	0.47±0.25	0.264
**CP grade**				0.667
A	221 (82.8)	156 (83.4)	65 (81.3)	
B	46 (17.2)	31 (16.6)	15 (18.8)	
**ALBI grade**				0.213
Grade 1	148 (55.4)	108 (57.8)	40 (50.0)	
Grade 2	114 (42.7)	77 (41.2)	37 (46.3)	
Grade 3	5 (1.9)	2 (1.1)	3 (3.8)	
**AFP(ng/mL)**				0.841
≤20	121 (45.3)	84 (44.9)	37 (46.3)	
>20	146 (54.7)	103 (55.1)	43 (53.8)	
**CEA(ug/L)**				0.369
≤5	222 (83.2)	158 (84.5)	64 (80.0)	
>5	45 (16.9)	29 (15.5)	16 (20.0)	
**CA19-9(ku/L)**				0.649
≤37	185 (69.3)	128 (68.5)	57 (71.3)	
>37	82 (30.7)	59 (31.6)	23 (28.8)	
**Tumor size (cm)**				0.256
≤5	136 (50.9)	91 (48.7)	45 (56.3)	
<5	131 (49.1)	96 (51.3)	35 (43.8)	
**Tumor number**				0.200
Single	182 (68.2)	123 (65.8)	59 (73.8)	
Multiple	85 (31.8)	64 (34.2)	21 (26.3)	
**MVI**				0.129
Absent	247 (92.5)	170 (90.9)	77 (96.3)	
Present	20 (7.5)	17 (9.1)	3 (3.8)	
**LNI**				0.483
Absent	239 (89.5)	169 (90.4)	70 (87.5)	
Present	28 (10.5)	18 (9.6)	10 (12.5)	
**AJCC TNM-8**				0.355
Ⅰ	117 (43.8)	83 (44.4)	34 (42.5)	
Ⅱ	53 (19.9)	35 (18.7)	18 (22.5)	
Ⅲ	61 (22.9)	47 (25.1)	14 (17.5)	
Ⅳ	36 (13.5)	22 (11.8)	14 (17.5)	

**Note:** HBV: hepatitis B virus; HCV: hepatitis C virus; ALT: alanine aminotransferase; ALB: albumin; TB: total bilirubin; AKP: alkaline phosphatase; γ-GT: γ-glutamyl transpeptidase; AAPR: Albumin-to-Alkaline Phosphatase Ratio; CP grade: Child-Pugh grade; ALBI grade: the albumin-bilirubin grade; AFP: Alfa-Fetoprotein; CEA: carcinoembryonic antigen; CA19-9: carbohydrate antigen 19-9; MVI: macroscopic vascular invasion; LNI: lymph node involvement; AJCC TNM-8: the 8th edition of American Joint Committee on Cancer TNM staging.

**Table 2:** Association between AAPR and other characteristics

Characteristics	Total (N=187)	AAPR≥0.43 (N=112)	AAPR<0.43 (N=75)	P value
**Age (years)**	52.6±11.7	51.0±11.2	55.1±12.2	0.019*
**Sex**				0.100
Male	146 (78.1)	92 (82.1)	54 (72.0)	
Female	41 (21.9)	20 (17.9)	21 (28.0)	
**Etiology**				0.183
HBV infection	141 (75.4)	90 (80.4)	51 (68.0)	
HCV infection	3 (1.6)	\	3 (4.0)	
Other	43 (23.0)	22 (19.6)	21 (28.0)	
**Liver cirrhosis (yes)**	131 (70.1)	78 (69.6)	53 (70.7)	0.881
**ALT (U/L)**	41.4±48.5	36.5±35.1	48.8±63.0	0.089
**ALB (g/L)**	41.4±4.3	42.5±4.1	39.7±4.2	<0.001*
**TB (umol/L)**	13.8±14.3	12.2±5.5	16.1±21.5	0.068
**AKP (U/L)**	93.9±37.8	73.1±15.7	124.8±40.1	<0.001*
**γ-GT (U/L)**				<0.001*
≤50	75 (40.1)	59 (52.7)	16 (21.3)	
>50	112 (59.9)	53 (47.3)	59 (78.7)	
**AARP**	0.51±0.21	0.62±0.20	0.34±0.07	<0.001*
**CP grade**				0.001*
A	156 (83.4)	102 (91.1)	54 (72.0)	
B	31 (16.6)	10 (8.9)	21 (28.0)	
**ALBI grade**				<0.001*
Grade 1	108 (57.8)	78 (69.6)	30 (40.0)	
Grade 2	77 (41.2)	34 (30.4)	43 (57.3)	
Grade 3	2 (1.1)	\	2 (2.7)	
**AFP (ng/mL)**				0.694
≤20	84 (44.9)	49 (43.8)	35 (46.7)	
>20	103 (55.1)	63 (56.3)	40 (53.3)	
**CEA (ug/L)**				0.027*
≤5	158 (84.5)	100 (89.3)	58 (77.3)	
>5	29 (15.5)	12 (10.7)	17 (22.7)	
**CA19-9 (ku/L)**				0.018*
≤37	128 (68.5)	84 (75.0)	44 (58.7)	
>37	59 (31.6)	28 (25.0)	31 (41.3)	
**Tumor size (cm)**				0.052
≤5	91 (48.7)	61 (54.5)	30 (40.0)	
<5	96 (51.3)	51 (45.5)	45 (60.0)	
**Tumor number**				0.675
Single	123 (65.8)	75 (67.0)	48 (64.0)	
Multiple	64 (34.2)	37 (33.0)	27 (36.0)	
**AJCC TNM-8**				0.066
Ⅰ	83 (44.4)	54 (48.2)	29 (38.7)	
Ⅱ	35 (18.7)	24 (21.4)	11 (14.7)	
Ⅲ	47 (25.1)	23 (20.5)	24 (32.0)	
Ⅳ	22 (11.8)	11 (9.8)	11 (14.7)	
**MVI**				0.122
Absent	170 (90.9)	105 (93.8)	65 (86.7)	
Present	17 (9.1)	7(6.3)	10 (13.3)	
**LNI**				0.450
Absent	169 (90.4)	103 (92.0)	66 (88.0)	
Present	18 (9.6)	9 (8.0)	9 (12.0)	

**Note:** HBV: hepatitis B virus; HCV: hepatitis C virus; ALT: alanine aminotransferase; ALB: albumin; TB: total bilirubin; AKP: alkaline phosphatase; γ-GT: γ-glutamyl transpeptidase; AAPR: Albumin-to-Alkaline Phosphatase Ratio; CP grade: Child-Pugh grade; ALBI grade: the albumin-bilirubin grade; AFP: Alfa-Fetoprotein; CEA: carcinoembryonic antigen; CA19-9: carbohydrate antigen 19-9; MVI: macroscopic vascular invasion; LNI: lymph node involvement; AJCC TNM-8: the 8th edition of American Joint Committee on Cancer TNM staging.

**Table 3:** Univariate and multivariate analyses for overall survival in the training cohort and validation cohort

Variable	Training cohort			Validation cohort		
	Univariate analysis	Multivariate analysis		Univariate analysis	Multivariate analysis	
	p-value	HR (95%CI)	p-value	p-value	HR (95%CI)	p-value
Age, years (≤65/>65)	0.091			0.768		
Sex (female/male)	0.285			0.789		
Hepatitis (no/yes)	0.651			0.337		
Liver cirrhosis (no/yes)	0.052			0.510		
ALT, U/L (≤40/>40)	0.057			0.821		
γ-GT, U/L (≤50/>50)	<0.001*	2.36(1.44-3.85)	0.001*	0.012*		
AARP (>0.40/≤0.40)	<0.001*	1.77(1.16-2.68)	0.007*	0.001*	2.19(1.06-4.51)	0.034*
CP grade (A/B)	0.020*			0.619		
ALBI grade (1/2/3)	0.120			0.432		
AFP, ng/mL (≤400/>400)	0.386			0.466		
CEA, ug/L (≤5/>5)	0.008*	1.69(1.03-2.76)	0.036*	0.073		
CA19-9, ku/L (≤37/>37)	<0.001*	1.65(1.08-2.54)	0.022*	0.056		
Tumor size, cm (≤5/>5)	<0.001*			<0.001*		
Tumor number (single /multiple)	0.020*			0.014*		
MVI (no/yes)	<0.001*			0.147		
LNI (no/yes)	<0.001*			0.028*		
AJCC TNM-8 (Ⅰ/Ⅱ/Ⅲ/Ⅳ)	<0.001*	1.75(1.43-2.14)	<0.001*	<0.001*	1.77(1.32-2.36)	<0.001*

**Note:** HR: hazard ratio; CI: confidence interval; ALT: alanine aminotransferase; γ-GT: γ-glutamyl transpeptidase; AAPR: Albumin-to-Alkaline Phosphatase Ratio; CP grade: Child-Pugh grade; ALBI grade: the albumin-bilirubin grade; AFP: Alfa-Fetoprotein; CEA: carcinoembryonic antigen; CA19-9: carbohydrate antigen 19-9; MVI: macroscopic vascular invasion; LNI: lymph node involvement; AJCC TNM-8: the 8th edition of American Joint Committee on Cancer TNM staging. *statistically significant.

**Table 4:** Comparison of the predictive performances of liver function assessment methods

Model	Training cohort				Validation cohort			
	C-index (95%CI)	AUC (95%CI)	LAT χ^2^	AIC	C-index (95%CI)	AUC (95%CI)	LAT χ^2^	AIC
AAPR	0.61 (0.55-0.67)	0.63 (0.56-0.70)	11.7	854	0.59 (0.51-0.67)	0.62 (0.51-0.73)	10.8	297
CP grade	0.57 (0.52-0.62)	0.55 (0.50-0.60)	4.6	861	0.48 (0.43-0.53)	0.50 (0.42-0.59)	0.2	308
ALBI grade	0.56 (0.50-0.62)	0.59 (0.52-0.66)	4.0	861	0.53 (0.45-0.61)	0.47 (0.35-0.58)	1.3	307

**Note:** AUC: area under the curve; C-index: concordance index; LAT χ2: likelihood ratio test χ^2^; AIC: Akaike information criteria; CI: confidence interval; AAPR: albumin-to-alkaline phosphatase ratio; CP grade: Child-Pugh grade; ALBI grade: the albumin-bilirubin grade.

**Table 5:** The predictive performances of the AAPR-based prognostic nomogram in comparison with the AJCC TNM-8

Model	Training cohort				Validation cohort			
	C-index (95%CI)	AUC (95%CI)	LAT χ^2^	AIC	C-index (95%CI)	AUC (95%CI)	LAT χ^2^	AIC
Nomogram	0.76 (0.71-0.81)	0.77 (0.70-0.83)	65.2	800	0.69 (0.60-0.78)	0.74 (0.63-0.86)	27.7	281
AJCC TNM-8	0.70 (0.65-0.75)	0.66 (0.58-0.73)	32.0	833	0.65 (0.56-0.74)	0.71 (0.61-0.82)	21.4	287

**Note:** AAPR: albumin-to-alkaline phosphatase ratio; AJCC TNM-8, the 8th edition of American Joint Committee on Cancer TNM staging; C-index: concordance index; AUC: area under the curve; LAT χ2: likelihood ratio test χ^2^; AIC: Akaike information criteria; CI: confidence interval.

## Abbreviations

HR: hazard ratio
CI: confidence interval
cHCC-CCA: combined hepatocellular and cholangiocarcinoma
HCC: hepatocellular carcinoma
CC: cholangiocarcinoma
HPCs: hepatic progenitor cells
WHO: World Health Organization
CP grade: Child-Pugh grade
CEA: carcinoembryonic antigen
CA19-9: carbohydrate antigen 19-9
AAPR: Albumin-to-Alkaline Phosphatase Ratio
ALB: albumin
AKP: alkaline phosphatase
OS: overall survival
CT: computerized tomography
MRI: magnetic resonance imaging
SD: standard deviation
ALBI grade: albumin-bilirubin grade
AUC: area under the curve
LAT: likelihood ratio test
AIC: Akaike information criteria
C-index: concordance index
DCA: decision curve analysis
HBV: hepatitis B virus
HCV: hepatitis C virus
AFP: Alfa-Fetoprotein
MVI: macroscopic vascular invasion
LNI: lymph node involvement
γ-GT: γ-glutamyl transpeptidase
ALT: alanine aminotransferase
TB: total bilirubin
EMT: epithelial mesenchymal transition
BCLC: the Barcelona Clinic Liver Cancer staging system
CLIP: the Cancer of the Liver Italian Program score
AJCC TNM-8: the 8th edition of American Joint Committee on Cancer TNM staging
