# Supplementary material for: Albumin-to-Alkaline Phosphatase Ratio is an Independent Prognostic Indicator in Combined Hepatocellular and Cholangiocarcinoma
Source: J Cancer. 2020 Jun 29;11(17):5177–86. doi: 10.7150/jca.45633 (PMC7378922; doi:10.7150/jca.45633)
Supplement: Supplementary file 1 — Supplementary figure S1. [file jcav11p5177s1.pdf]

Figure S1 X-tile analyses regarding to OS by using patient data to determine the optimal cut-off value for AAPR. OS: overall survival. AAPR: albumin-to-alkaline phosphatase ratio.

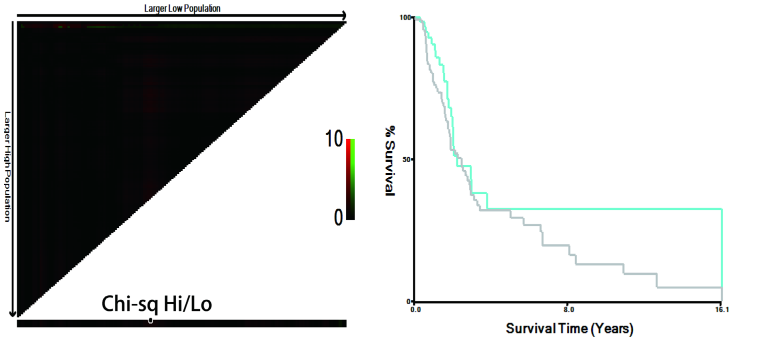

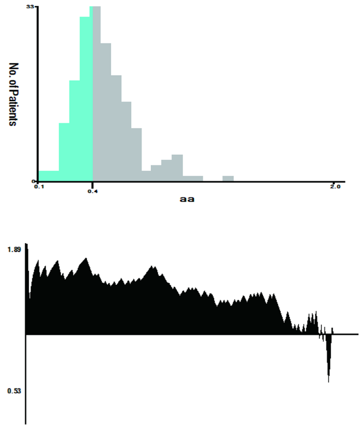

Subpopulation Cutpoints:

Pt No	% Total	Events	Rate	Rank	Range
75	40.11	23	30.67	0 to 71	0.09 thru 0.43
112	59.89	64	57.14	72 to 175	0.43 thru 1.95
187	100.00	87	46.52	0 to 175	0.09 thru 1.95
